# The effect of rapid maxillary expansion on the upper airway’s aerodynamic characteristics

**DOI:** 10.1186/s12903-021-01488-1

**Published:** 2021-03-17

**Authors:** Xin Feng, Yicheng Chen, Kristina Hellén-Halme, Weihua Cai, Xie-Qi Shi

**Affiliations:** 1 Department of Clinical Dentistry, Faculty of Medicine, University of Bergen, Årstadveien 19, 5009 Bergen, Norway; 2 School of Energy Science and Engineering, Harbin Institute of Technology, Xi Da Zhi Street, Nangang, 150001 Harbin People’s Republic of China; 3 Department of Oral and Maxillofacial Radiology, Faculty of Odontology, Malmö University, 205 06, Malmö, Sweden; 4 School of Energy and Power Engineering, Northeast Electric Power University, Changchun Road 169, Changchun, 132012 Jilin People’s Republic of China

**Keywords:** Computational fluid dynamics, Upper airway, Adenoid hypertrophy, Rapid maxillary expansion

## Abstract

**Background:**

The effect of rapid maxillary expansion (RME) on the upper airway (UA) has been studied earlier but without a consistent conclusion. This study aims to evaluate the outcome of RME on the UA function in terms of aerodynamic characteristics by applying a computational fluid dynamics (CFD) simulation.

**Methods:**

This retrospective cohort study consists of seventeen cases with two consecutive CBCT scans obtained before (T0) and after (T1) RME. Patients were divided into two groups with respect to patency of the nasopharyngeal airway as expressed in the adenoidal nasopharyngeal ratio (AN): group 1 was comprised of patients with an AN ratio < 0.6 and group 2 encompassing those with an AN ratio ≥ 0.6. CFD simulation at inspiration and expiration were performed based on the three-dimensional (3D) models of the UA segmented from the CBCT images. The aerodynamic characteristics in terms of pressure drop (ΔP), maximum midsagittal velocity (V_ms_), and maximum wall shear stress (P_ws_) were compared by paired t-test and Wilcoxon test according to the normality test at T0 and T1.

**Results:**

The aerodynamic characteristics in UA revealed no statistically significant difference after RME. The maximum V_ms_ (m/s) decreased from 2.79 to 2.28 at expiration after RME (*P* = 0.057).

**Conclusion:**

The aerodynamic characteristics were not significantly changed after RME. Further CFD studies with more cases are warranted.

## Background

Adenoid hypertrophy (AH) is a common cause of upper airway (UA) obstruction in children and adolescents. Considerable variation in AH prevalence, ranging from 27 to 80%, has been reported between countries and ages [1]. AH may cause several health issues including mouth breathing, snoring, asthma, speech problems, and obstructive sleep apnoea [2, 3]. To diagnose the degree of AH, Fujioka proposed calculating an adenoidal nasopharyngeal (AN) ratio by measuring adenoid thickness and nasopharyngeal width on lateral radiography, a common procedure in clinics [4, 5]. An AN ratio of more than 0.6 indicates a suspected nasal obstruction [2]. Otolaryngologists usually suggest an adenoidectomy to treat severe nasal obstruction, and this has been shown to positively affect volume expansion in the nasopharynx and improve nasal breathing. However, a noticeable recurrence of nasal obstruction after adenoidectomy has been reported [6]. In order to achieve a stable outcome after an adenoidectomy, several adjunctive treatments have been suggested for patients with specific symptoms including turbinoplasty, adenotonsillectomy, and rapid maxillary expansion (RME) [7–9].

AH may cause abnormal craniofacial development such as a short cranial base, long face, small and narrow maxilla, and mandibular retrusion [10–12]. Some orthodontists suggest that RME may have the potential to reduce nasal obstruction by opening the midsagittal suture, widening the maxillary arch, and increasing nasal space [9, 13, 14].

RME’s possible effect on nasal obstruction has been evaluated by several methods including rhinomanometry, acoustic rhinometry, polysomnography (PSG), cephalometric radiographs, cone beam computed tomography (CBCT) and computed tomography (CT), but with inconsistent conclusions [15, 16]. Laboratory-based PSG is considered the gold standard for diagnosing obstructive sleep apnoea, as it provides quantitative parameters to evaluate respiratory function such as the apnoea–hypopnea index [17]. However, it also has limited availability and is relatively expensive and time consuming, which could be inconvenient for children and their families. Therefore, researchers have been searching for alternative methods to evaluate the respiratory function of UA. For example, De Backer et al. [18] introduced computational fluid dynamics (CFD) as a diagnostic tool to observe the outcome of mandibular advancement devices when treating sleep-related breathing disorders and found that CFD models precisely capture UA’s aerodynamic characteristics. Moreover, the CFD results show a higher correlation with clinical symptoms than volumetric measurements on CT images.

The CFD method is a well-established technique that has been widely used in mechanical engineering, yet it is quite new to flow analysis in medicine. Based on a three-dimensional (3D) structure segmented from CBCT, CT, or magnetic resonance imaging (MRI), the CFD simulates and calculates the flow of gases or fluids and their interactions with the surrounding surfaces as defined by boundary conditions. At a given inlet pressure, the shape and boundary condition of a pipe-like UA would theoretically determine the aerodynamic characteristics in terms of pressure, velocity, and wall shear stress. The application of CFD in dentistry is nevertheless sparse. Few previous studies have shown that CFD could be applied to evaluate the outcome of mandible advanced devices on respiratory function [18, 19]. Regarding the effect of RME on airflow within the UA, Iwasaki et al. observed an improvement in nasal cavity obstruction [20] and a decrease in pharyngeal airway pressure after RME [21]. More clinical evidence on the changes of UA following RME is, however, needed to enhance and benefit individual treatment planning for patients with a narrow maxilla and enlarged adenoid.

In this study, we aim to evaluate the effect of RME on airflow within the UA by investigating the aerodynamic characteristics that result from applying CFD simulation. The null hypothesis is that RME has a positive effect on UA ventilation.

## Methods

This is a retrospective cohort study. All methods were carried out in accordance with the declaration of research involving human subjects and the regional ethical and scientific guidelines in Vestland region, Norway. Data for all patients who had undergone RME were retrospectively collected at the Department of Orthodontics (Stomatological hospital, Dalian, China) between January 2013 and December 2016. The inclusion criteria were patients younger than 15 years old who had both pre- and post-CBCT scans due to orthodontic indication. The pre-RME CBCTs were taken within seven days prior to fixing the expander (T0) and the post-RME CBCTs at the removal of expanders (T1). The exclusion criteria were severe abnormalities of maxillofacial tissue, previous surgery on skeletal and soft tissue related to respiration, and previous orthodontic or orthopaedic treatment. Eventually, 17 patients (mean age 12.2 ± 1.3 years, 11 male/6 female) were eligible for inclusion in the study. An experienced radiologist viewed all CBCT scans and ensured that the images were qualified to construct 3D models of the UA.

### Maxillary expansion protocol

A fixed Hyrax expander was used for RME, banded to the maxillary first premolars and first molars. The patient, or their guardian, rotated the expansion screw twice a day at home and a clinical check-up was performed by orthodontists once a week. The expansion was terminated when the occlusal aspect of the maxillary lingual cusps of the upper first molars contacted the occlusal aspect of the vestibular cusp of the mandibular first molars. After achieving the desired expansion, the expander remained in place for 5.2 ± 1.7 months to stabilise the expansion.

### CFD simulation

**Fig. 1:**
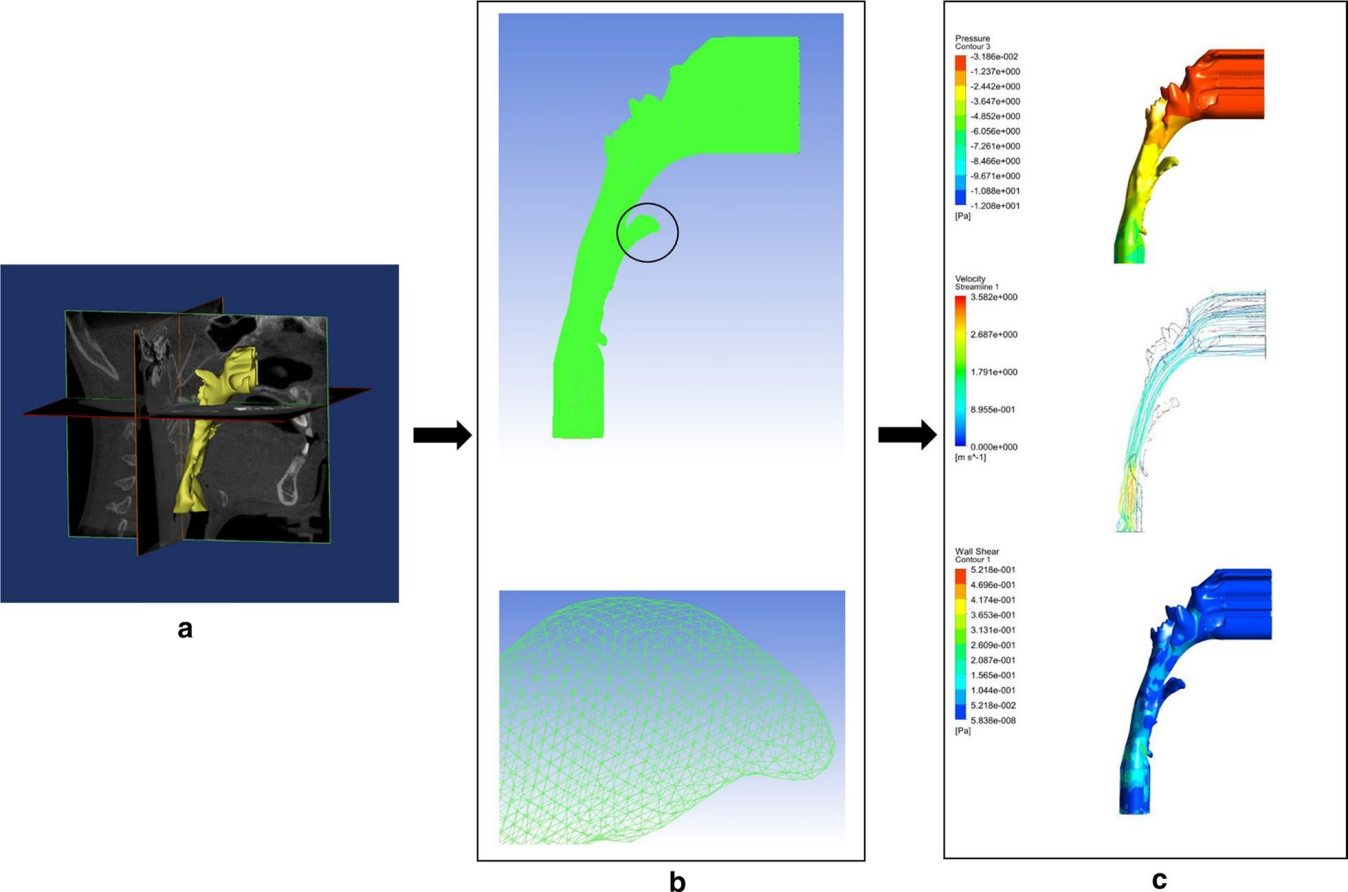
The procedure of CFD modeling and simulation. **a** CBCT segmentation, **b** Mesh generation and detailed zoom, **c** CFD simulation results: airflow pressure contour, velocity streamline, and wall shear stress contour

Figure 1 demonstrates the stepwise procedure of the CFD modelling and simulation, including 3D segmentation, mesh generation, and aerodynamic results.

### CBCT imaging

**Fig. 2:**
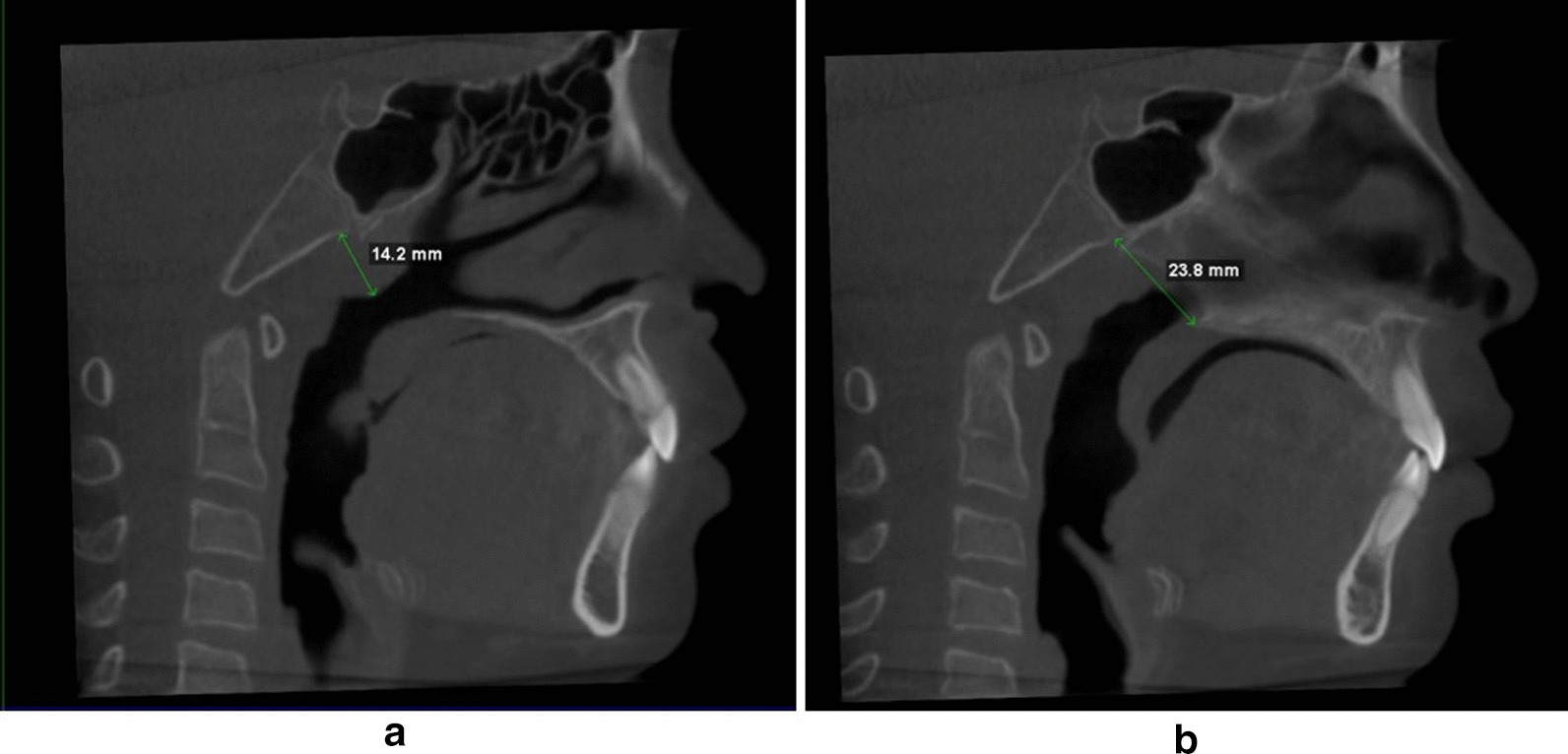
The measurement of AN ratio on CBCT images. **a** A, perpendicular distance from the maximal convexity of the adenoid identifying by scrolling through the sagittal slice that showed maximal convexity of the adenoid (where the intersecting axial view also showed maximal convexity) to the anterior margin of the basiocciput. **b** N, distance between the posterosuperior point of the hard palate and the anteroinferior point of the spheno-occipital synchondrosis on the mid-sagittal plane

The examination protocol of CBCT scans was as follows: field of view (FOV) 16 × 13 cm; tube potential 120 kVp and tube current 5 mA; scanning time 14.7 s (3D eXam; KaVo, Biberach an der Riss, Germany). The voxel size was set at 0.2 mm, and the contrast resolution had a 14-bit depth. All CBCT examinations were performed according to the standardised clinical routine, i.e. with the Frankfort horizontal plane parallel to the floor, teeth in maximum intercuspation, and peaceful nasal breathing without swallowing. We divided the 17 patients into two groups according to the AN ratio at baseline (T0): group 1 was comprised of individuals with an AN ratio < 0.6 and group 2 encompassing those with an AN ratio ≥ 0.6. The measurements of AN ratios were performed aiming to present the geometric obstruction status of the UA following Fujioka’s method [4]. A and N indicated the adenoid thickness and nasopharyngeal width, respectively (Fig. 2). The CBCT images were imported to MIMICS software (Materialise Mimics 23.0, Belgium) in the digital imaging and communications in medicine (DICOM) format for later analysis. To segment the 3D UA, one author (XF) orientated the CBCT image. An appropriate threshold was set from − 1024 to − 500 to involve the UA without defection [22], which was called a “mask”. The superior boundary was defined on the mask as perpendicular to the horizontal plane through the most posterior point of middle turbinate in the sagittal view; the inferior boundary was parallel to the horizontal plane through the most anterior–inferior point of cervical vertebra 4. The 3D UA was then calculated from the defined mask. The superior and inferior boundaries were extended by 20 mm to avoid flow reversing [23]. The extended 3D model was used to create a surface model for further mesh generation.

### Mesh generation

Mesh generation is the practice of creating a mesh by computer algorithms. The continuous geometric UA space may be subdivided into discrete geometric cells. Mesh cell is the fundamental element of the reconstructed space that contains a local approximation of aerodynamic characteristics, which will be used for a later calculation. We chose tetrahedral and prismatic cells to construct the main body and boundary layer of the UA (ANSYS, Inc., Canonsburg, Pennsylvania). Each UA mesh had five boundary layers and an average of 2 million elements. The inlet and outlet of UA were defined at the extended superior and inferior boundary, as earlier described.

### Aerodynamic analysis

ANSYS Fluent (ANSYS, Inc.) was applied to simulate the airflow of UA, and the SST κ-ω model was used to calculate the aerodynamic characteristics of UA. The wall of UA was defined as no-slip, stationary, and rigid, and the temperature and density of air were set as fixed [24]. In the inspiratory phase, the inlet was set with pressure 0 Pa and the outlet a flow rate of − 200 mL/s [20]. The corresponding values were − 200 mL/s and 0 Pa at inlet and outlet for the expiratory phase. Over 2000 iterations were performed to ensure the resulting residuals were less than 10^–6^. A radiologist (XF) performed all the simulations under the technical supervision of a fluid engineer (YCC). The CFD simulations were repeated six months later on ten randomly selected cases by the same operator (XF).

### Data analyses

**Fig. 3:**
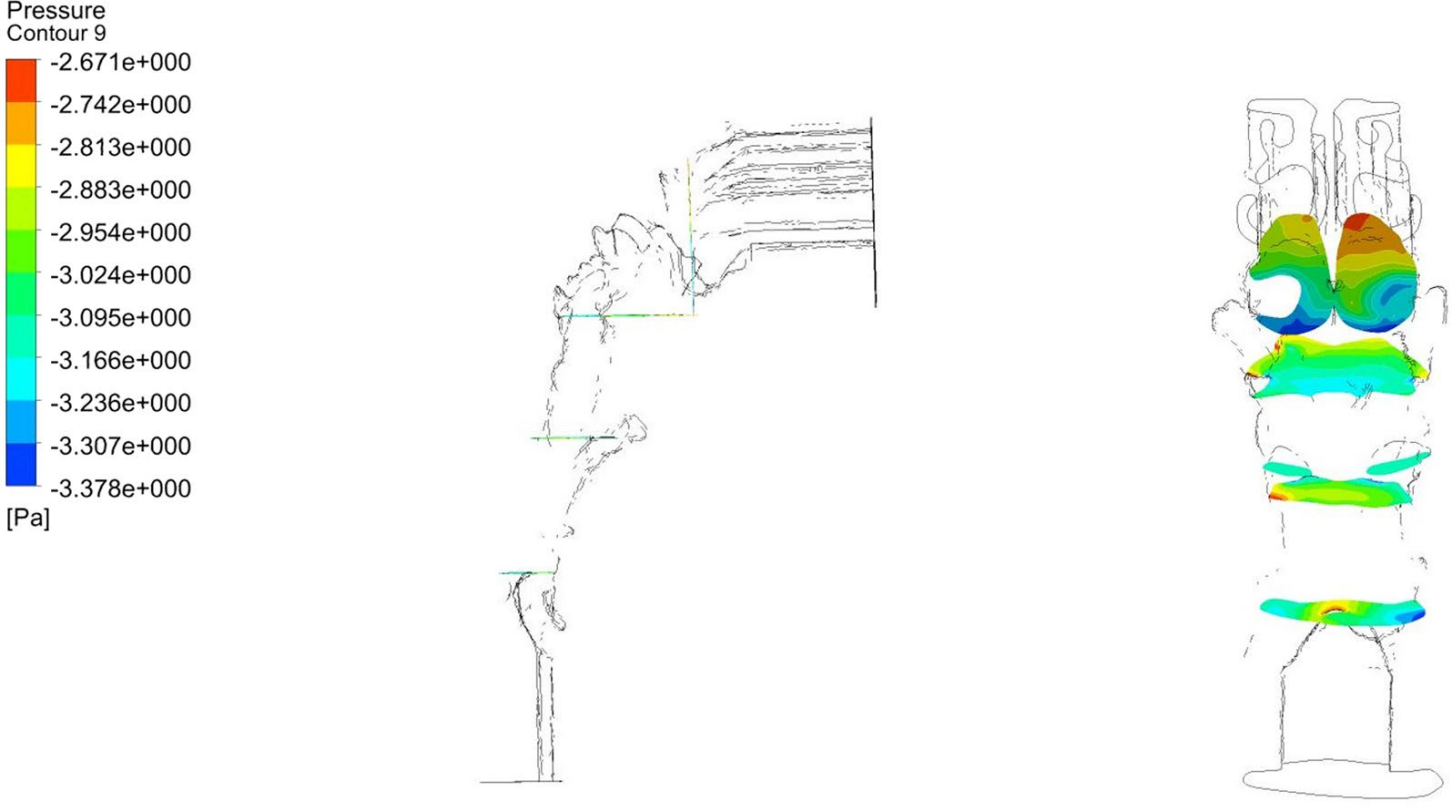
Description of the pressure of 4 planes defined on the CFD model. Definition of the four planes in the sagittal view: plane 1, paralleled the inlet plane through the posterior point of middle turbinate; plane 2, paralleled the outlet plane through the inferior point of plane 1; plane 3, paralleled the outlet plane through the tip of the soft palate; plane 4, paralleled the outlet plane through the tip of the epiglottis.The right graph shows the distribution of the pressure of each plane in the posterior view

We calculated the aerodynamic characteristics at inspiratory and expiratory phases, including mean pressure at the four planes defined on UA (Fig. 3). The parameters included are the pressure drop (ΔP) from plane 1 to plane 4, the maximum mid-sagittal velocity (V_ms_), and maximum wall shear stress (P_ws_) at T0 and T1. Data were processed using the IBM-SPSS version 25.0 (IBM, New York, NY, USA). Significance was set at *p* less than 0.05. Statistical tests for normality were conducted for all variables. Accordingly, paired t-test or Wilcoxon test was used to compare the changes of the aerodynamic characteristics between T0 and T1. Intraclass Correlation Coefficient (ICC) was applied to test the consistency of the CFD simulations.

## Results

The comparison of aerodynamic characteristics in terms of ΔP, the maximum V_ms_ and maximum P_ws_ of the UA between before (T0) and after (T1) RME were shown in Table 1. The ICC ranged between 0.787 and 1 for all measurements indicating the high repeatability of CFD method.

**Table 1:** Comparison of pressure drop (ΔP), maximum midsagittal velocity (V_ms_), and maximum wall shear stress (P_ws_) at inspiration and expiration before (T0) and after (T1) rapid maxillary expansion (n = 17)

	T0		T1		T0 versus T1	
	Mean	SD	Mean	SD	*p* value	
					Paired t test	Wilcoxon test
*Inspiration*						
ΔP (Pa)	− 4.00	1.87	− 4.36	2.45	0.549	
Maximum V_ms_ (m/s)	2.48	0.70	2.43	0.92		0.906
Maximum P_ws_ (Pa)	1.29	1.24	1.03	1.32		0.163
*Expiration*						
ΔP (Pa)	2.96	2.56	2.81	2.43		0.943
Maximum V_ms_ (m/s)	2.79	1.09	2.28	0.82	0.057	
Maximum P_ws_ (Pa)	1.63	1.85	0.93	0.71		0.381

**Fig. 4:**
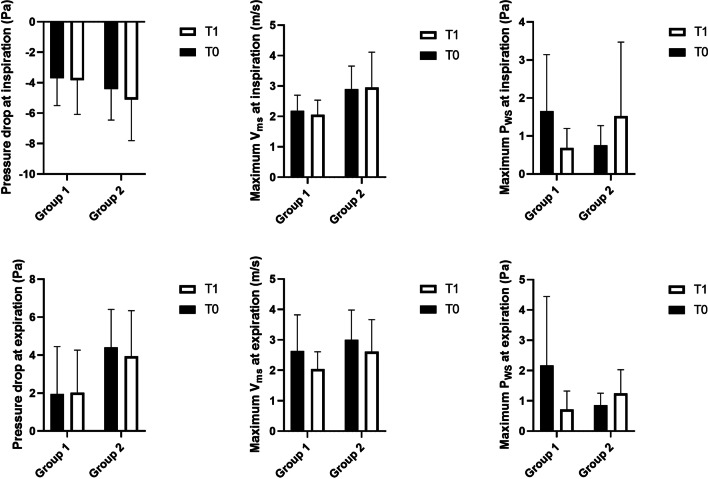
Expression of the aerodynamic characteristics in terms of mean, SD for the two groups T0 and T1

Among the 17 patients, ten patients were classified in group 1 (mean age 11.9 ± 1.3 years); seven patients in group 2 (mean age 12.6 ± 1.3 years). Figure 4 illustrates the distributions of the aerodynamic variables for the two groups at T0 and T1 graphically. It demonstrates that group 2 has higher mean ΔP and mean V_ms_ than group 1 at both inspiration and expiration regardless of T0 or T1; whereas the maximum P_ws_ shows the opposite trend being lower for group 2.

Due to the limited number of cases, group 1 and group 2 were merged when performing the statistical analysis on the effect of RME. Except for ΔP at inspiration, all the other aerodynamic parameters decrease after RME treatment (Table 1). However, none of the changes is statistically significant, of which the V_ms_ (m/s) drop (2.79–2.28) at expiration is close to being significant (*p* = 0.057).

## Discussion

### CFD simulation

In the engineering field, the pressure drop is defined as the pressure difference between two points of a fluid carrying network, which occurs when frictional forces, caused by the resistance to flow, interact with fluid as it flows through the tube. Applying this concept to airflow passing through the UA, the pressure drops when facing physical force caused by morphological changes. Furthermore, the maximum V_ms_ may be altered following UA morphological changes. Faramarzi et al. evaluated the aerodynamics of the nasal cavity in a patient with septal perforation and found higher velocity at areas with higher pressure drop [25]. Regarding wall shear stress, it expresses the force per unit area exerted by the wall on the fluid in a direction on the local tangent plane [26]. The maximum P_ws_ locates mostly at a constricted area [27]. A successful expansion of maxilla suture would hypothetically increase the UA space, resulting in declines in ΔP, maximum V_ms_ and maximum P_ws_.

In the present study, CFD simulation was applied to elucidate the aerodynamic characteristics of the UA before and after RME. The null hypothesis was rejected, i.e. RME does not have a positive effect on UA ventilation. We failed to observe any statistically significant change in airflow characteristics after RME despite overall declines of ΔP, maximum V_ms_ and maximum P_ws_. The difference in V_ms_ after RME (2.79–2.28) at expiration is nearly significant (*p* = 0.057). This finding is in line with previous reports where the airflow resistance at expiration was found to be closely related to obstructive severity [28, 29]. Also, Chen et al. reported that patients with obstructive sleeping problem had a higher airflow resistance during the expiratory phase than the healthy subjects applying by CFD simulation [30].

The effect of RME on aerodynamics has been investigated sparsely. In contrast to the present study, Iwasaki et al. found significant changes in aerodynamic characteristics in the nasal cavity after RME [21]. This may imply that the RME mainly increases the maxilla width in the transverse direction and the skeletal boundary of nasal cavity was directly extended following with the expanded maxilla [20]. The pharyngeal part of UA is surrounded by multiple soft tissues and located posteriorly to the maxilla. Thus the positive effect of RME on UA is more notable in the nasal cavity than the lower UA region. More cases are needed to detect possible effect and to increase the power of the applied statistics.

Enlarged adenoid is a common cause of nasal obstruction in children. Knowledge of aerodynamics in this group of patients would help understand the disease mechanism, assist diagnostics and evaluate treatment outcomes. Due to the small sample size, we did not perform statistical analysis on the effect of RME for each individual group. Nevertheless, ΔP and maximum V_ms_ seemed to be lower in group 1 as compared to group 2 regardless of T0 or T1 (Fig. 4), indicating air resistance in UA seemed to be higher in patients with enlarged adenoids. However, we are puzzled by the results of the maximum P_ws_.

It has been reported that one of the most restricted areas in UA was located at the velopharynx where the maximum P_ws_ and a pharyngeal jet were observed [31, 32]. In our case, we speculate that an “adenoid jet” might have occurred when airflow passing through the enlarged adenoid (group 2). The high-speed adenoid jet might have caused strong vortexes and a complex recirculation resulting in a retarded downstream velocity gradient near UA’s wall and thus a reduced maximum P_ws_. Consequently, the maximum P_ws_ was lower in group 2 than group 1 at T0. After RME the adenoid jet may be weakened, resulting in a increased maximum P_ws_ in group 2. However, due to limited cases and the diverse airflow characteristics in group 2, random effect can not be excluded. Therefore, more cases with severely enlarged adenoids are needed to confirm our assumption.

CFD is a valuable tool for investigating the aerodynamic characteristics of the UA for better understanding the complex airflow ventilation related to UA morphology. At present, the simulation procedure is not entirely automatic and thus very time consuming. Part of the 3D segmentation and mesh generation needs to be performed manually due to the irregular anatomic structure of the UA. This may be the cause for the limited number of samples in the available CFD studies [19, 33, 34]. However, we do believe, with the help of artificial intelligence the CFD simulation procedure could be simplified and less time consuming in the near future.

### Clinical implications

The CFD method makes the aerodynamic characteristics within the UA visible. However, due to the intrinsic nature of a retrospective study design, the lack of clinical otolaryngologic examination makes it difficult to conclude whether RME would affect the airflow condition. Nevertheless, the enlarged adenoid may influence the UA’s ventilation. Further perspective study is warranted to identify the specific patients who may benefit from the RME.

## Conclusions

The aerodynamic characteristics were not significantly changed after RME. Further CFD studies with more cases are warranted.

## Data Availability

All data used and/or analysed during the current study are available from the corresponding author on reasonable request.
